# Supplementary material for: Therapeutic Effects of a Novel Aptamer on Coronaviral Infection-Induced Lung Injury and Systemic Inflammatory Responses
Source: Cells. 2024 Feb 28;13(5):422. doi: 10.3390/cells13050422 (PMC10931054; doi:10.3390/cells13050422)
Supplement: Supplementary file 1 [file cells-13-00422-s001.zip › cells-2855923-supplementary.pptx]

## Slide 1
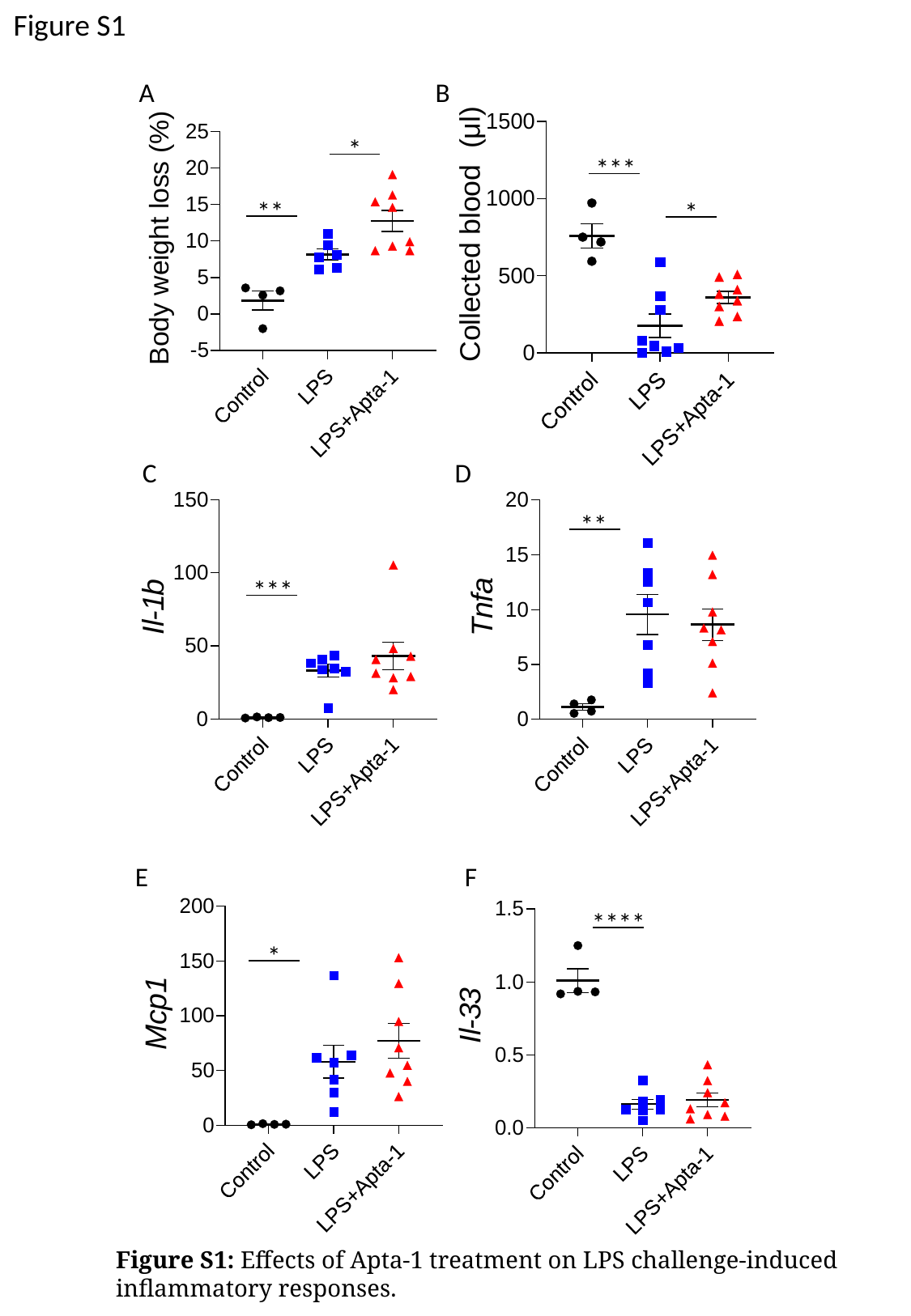

Figure S1
A
B
*
***
**
*
C
D
**
***
E
F
****
*
Figure S1: Effects of Apta-1 treatment on LPS challenge-induced inflammatory responses.

## Slide 2
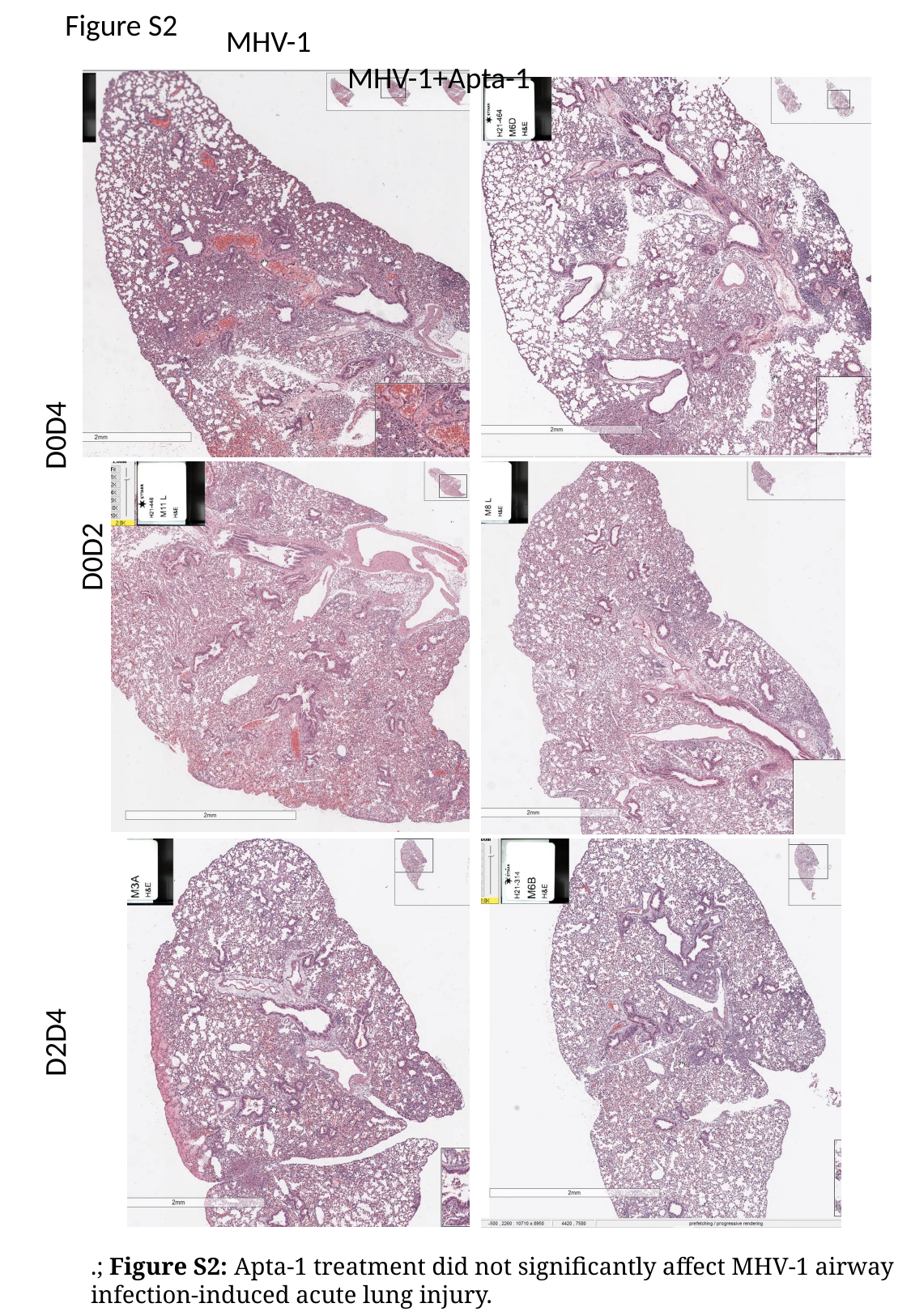

MHV-1					MHV-1+Apta-1
D2D4					D0D4						D0D2
Figure S2
.; Figure S2: Apta-1 treatment did not significantly affect MHV-1 airway infection-induced acute lung injury.

## Slide 3
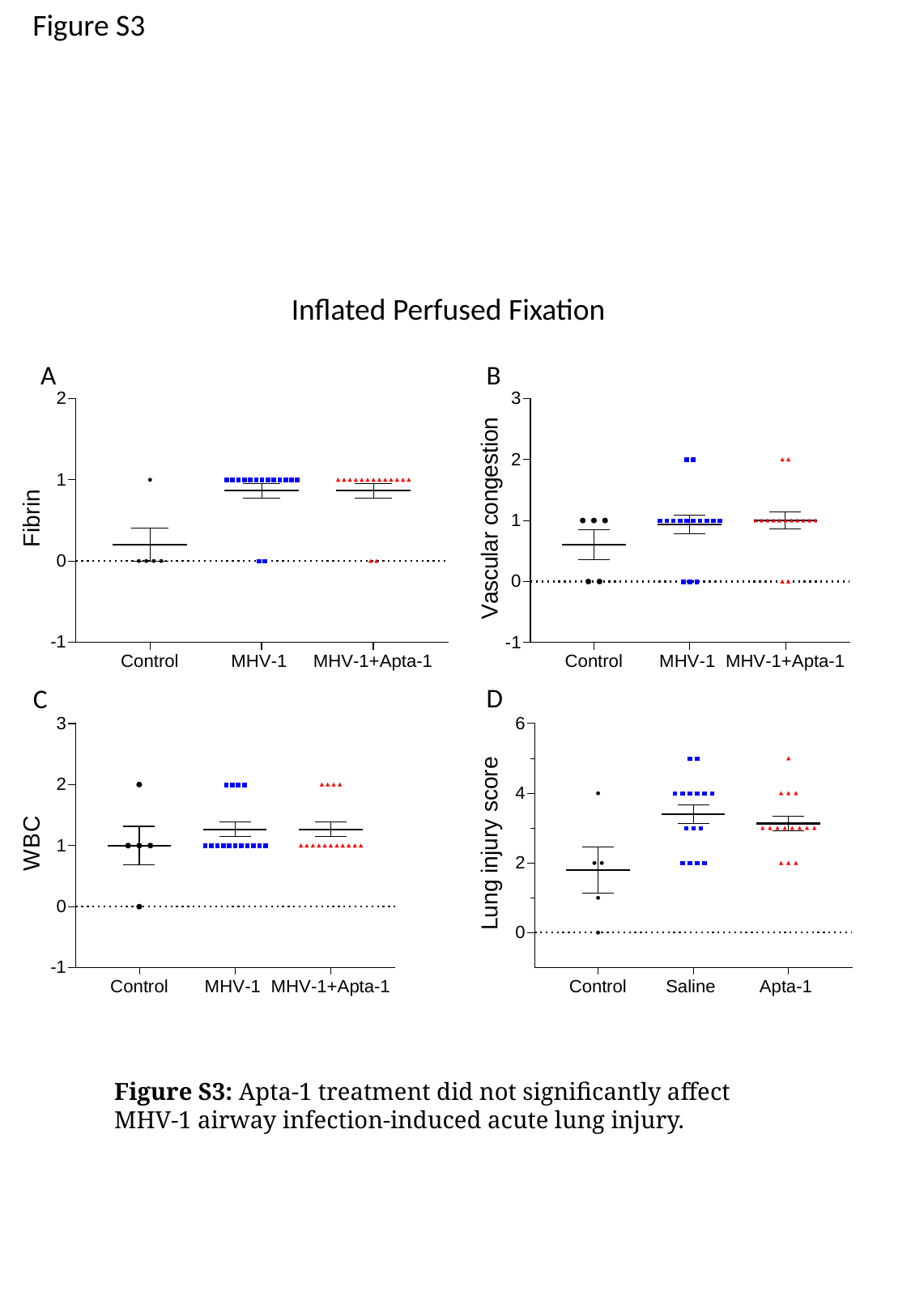

Figure S3
Inflated Perfused Fixation
A
B
D
C
Figure S3: Apta-1 treatment did not significantly affect MHV-1 airway infection-induced acute lung injury.

## Slide 4
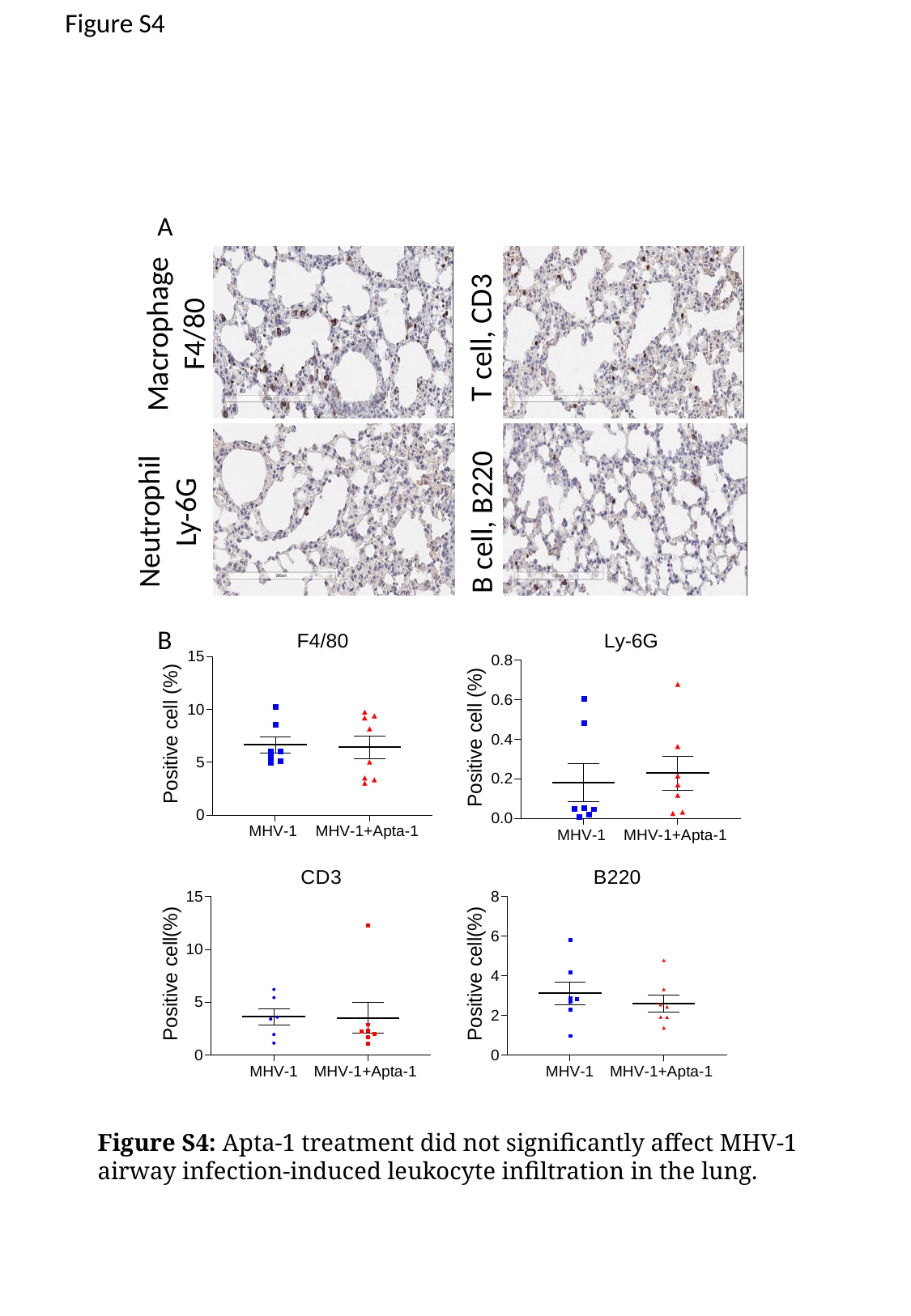

Figure S4
A
Macrophage
F4/80
T cell, CD3
Neutrophil
 Ly-6G
B cell, B220
B
Figure S4: Apta-1 treatment did not significantly affect MHV-1 airway infection-induced leukocyte infiltration in the lung.

## Slide 5
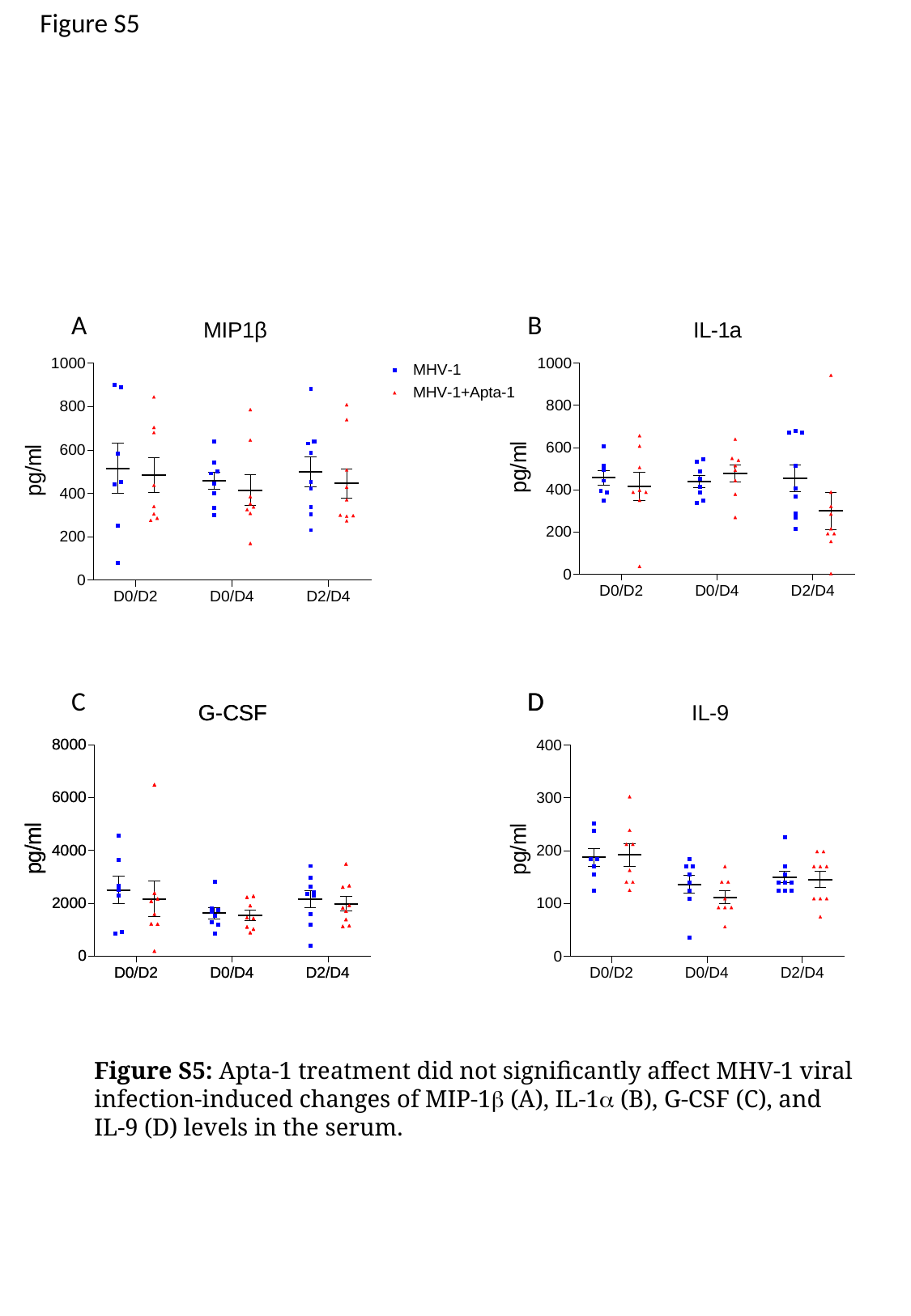

Figure S5
A
B
C
D
D
Figure S5: Apta-1 treatment did not significantly affect MHV-1 viral infection-induced changes of MIP-1β (A), IL-1α (B), G-CSF (C), and IL-9 (D) levels in the serum.

## Slide 6
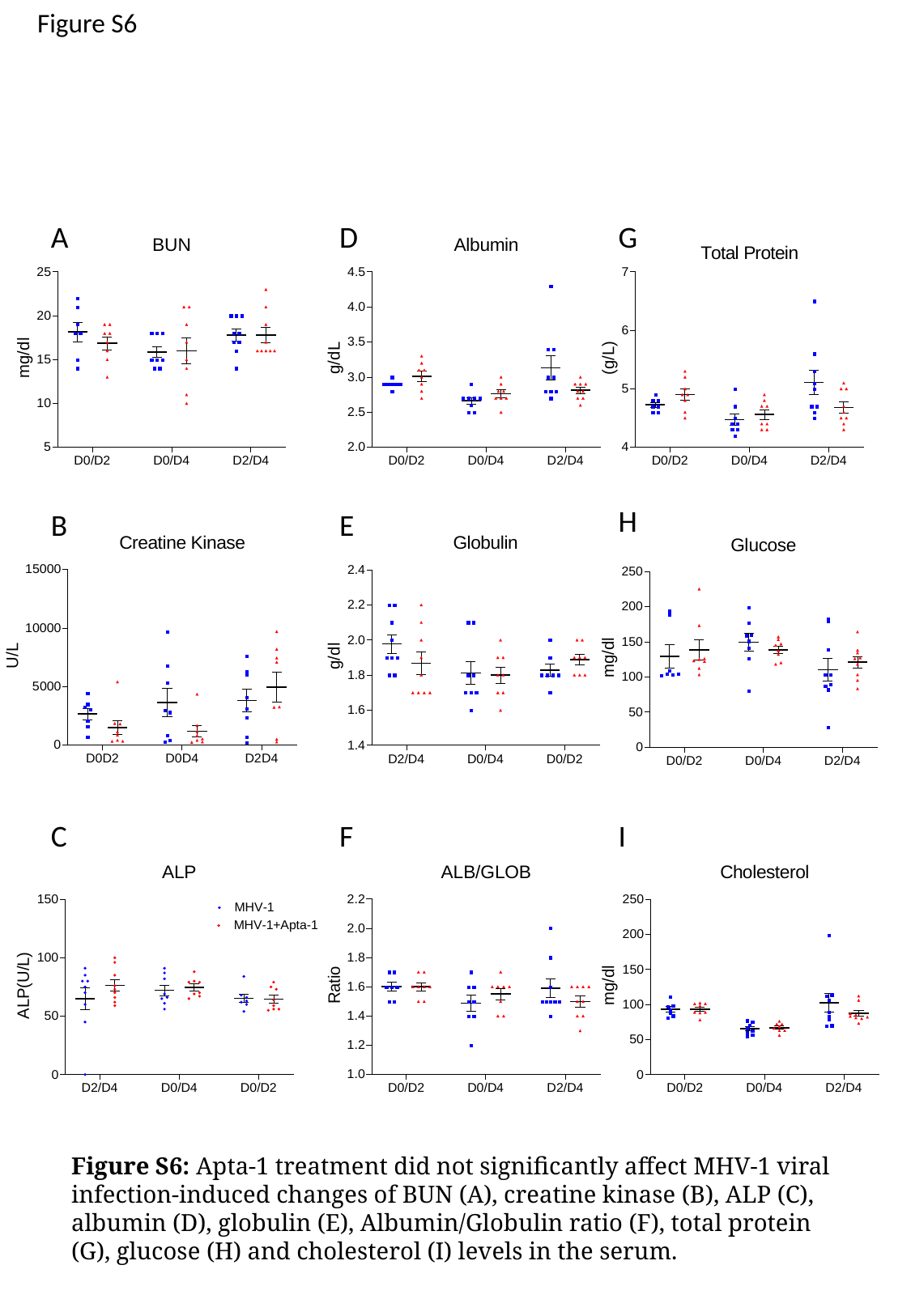

Figure S6
A
D
G
H
B
E
C
F
I
Figure S6: Apta-1 treatment did not significantly affect MHV-1 viral infection-induced changes of BUN (A), creatine kinase (B), ALP (C), albumin (D), globulin (E), Albumin/Globulin ratio (F), total protein (G), glucose (H) and cholesterol (I) levels in the serum.

## Slide 7
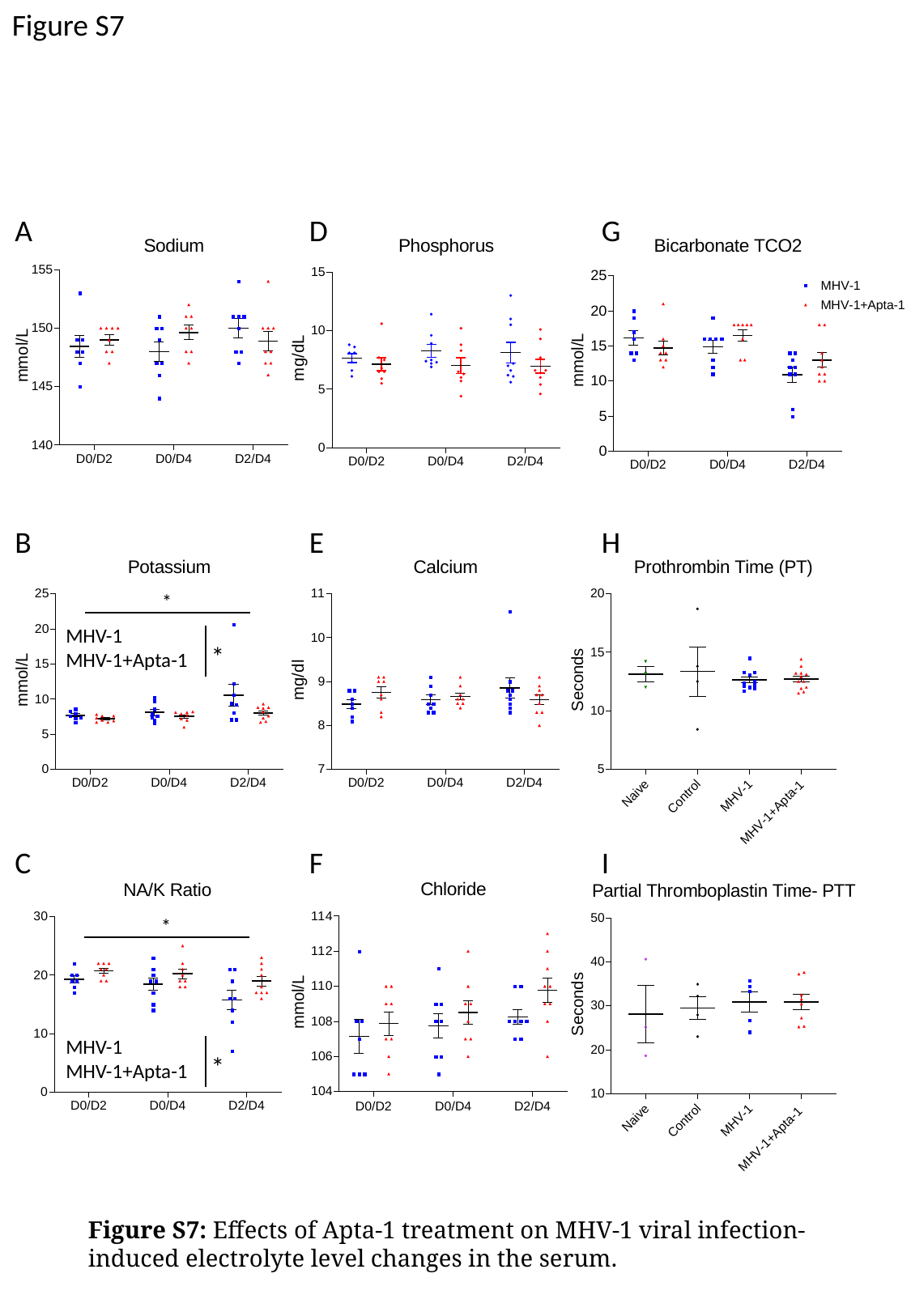

Figure S7
A
D
G
B
E
H
*
MHV-1
MHV-1+Apta-1
*
C
F
I
*
MHV-1
MHV-1+Apta-1
*
Figure S7: Effects of Apta-1 treatment on MHV-1 viral infection-induced electrolyte level changes in the serum.

## Slide 8
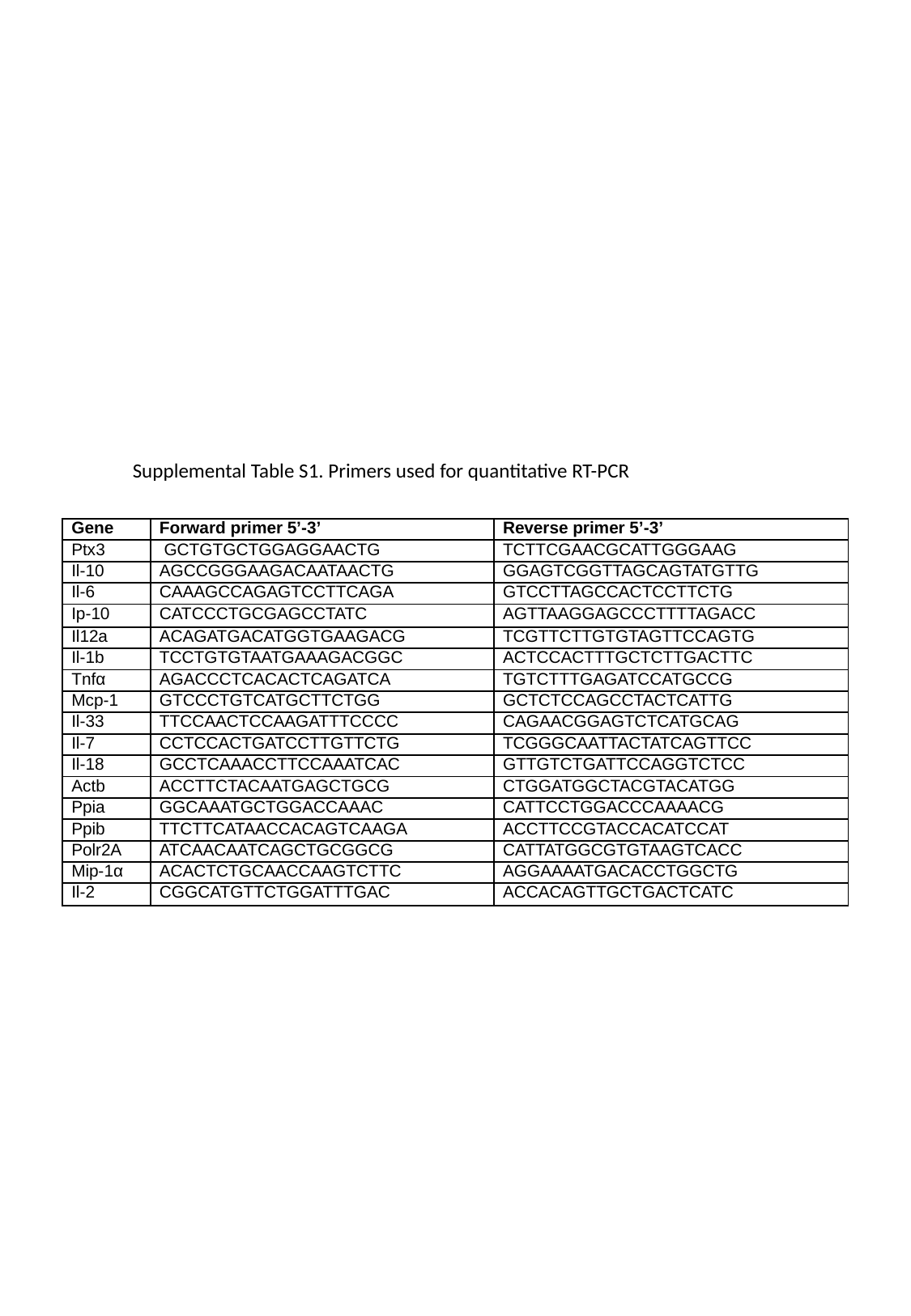

Supplemental Table S1. Primers used for quantitative RT-PCR
| Gene | Forward primer 5’-3’ | Reverse primer 5’-3’ |
| --- | --- | --- |
| Ptx3 | GCTGTGCTGGAGGAACTG | TCTTCGAACGCATTGGGAAG |
| Il-10 | AGCCGGGAAGACAATAACTG | GGAGTCGGTTAGCAGTATGTTG |
| Il-6 | CAAAGCCAGAGTCCTTCAGA | GTCCTTAGCCACTCCTTCTG |
| Ip-10 | CATCCCTGCGAGCCTATC | AGTTAAGGAGCCCTTTTAGACC |
| Il12a | ACAGATGACATGGTGAAGACG | TCGTTCTTGTGTAGTTCCAGTG |
| Il-1b | TCCTGTGTAATGAAAGACGGC | ACTCCACTTTGCTCTTGACTTC |
| Tnfα | AGACCCTCACACTCAGATCA | TGTCTTTGAGATCCATGCCG |
| Mcp-1 | GTCCCTGTCATGCTTCTGG | GCTCTCCAGCCTACTCATTG |
| Il-33 | TTCCAACTCCAAGATTTCCCC | CAGAACGGAGTCTCATGCAG |
| Il-7 | CCTCCACTGATCCTTGTTCTG | TCGGGCAATTACTATCAGTTCC |
| Il-18 | GCCTCAAACCTTCCAAATCAC | GTTGTCTGATTCCAGGTCTCC |
| Actb | ACCTTCTACAATGAGCTGCG | CTGGATGGCTACGTACATGG |
| Ppia | GGCAAATGCTGGACCAAAC | CATTCCTGGACCCAAAACG |
| Ppib | TTCTTCATAACCACAGTCAAGA | ACCTTCCGTACCACATCCAT |
| Polr2A | ATCAACAATCAGCTGCGGCG | CATTATGGCGTGTAAGTCACC |
| Mip-1α | ACACTCTGCAACCAAGTCTTC | AGGAAAATGACACCTGGCTG |
| Il-2 | CGGCATGTTCTGGATTTGAC | ACCACAGTTGCTGACTCATC |
